# Homeostatic glucocorticoid signaling in airway smooth muscle: A roadmap to asthma pathogenesis

**DOI:** 10.3389/fendo.2022.1077389

**Published:** 2023-01-04

**Authors:** Michael M. Grunstein

**Affiliations:** Perelman School of Medicine, University of Pennsylvania, Philadelphia, PA, United States

**Keywords:** asthma, airway smooth muscle, homeostasis, cytokines, beta2-adrenergic desensitization, mitogen-activated protein kinases, autologous glucocorticoid signaling

## Abstract

Homeostasis is the self-regulating process by which the body maintains internal stability within a narrow physiological range (i.e., “normality”) as it dynamically adjusts to disruptive influences. Thus, whereas homeostasis maintains bodily health, disrupted homeostasis at the tissue or systemic level leads to disease. Airway smooth muscle (ASM) is the pivotal site of disrupted homeostasis in asthma. While extensive research has greatly expanded our understanding of ASM behavior under pro-asthmatic conditions, the cellular signaling mechanisms that underlie ASM homeostasis under these conditions remain elusive. Based on a broad collection of published studies, a homeostasis mechanism intrinsic to ASM and exhibited under inflammatory and non-inflammatory pro-asthmatic conditions is identified herein. Central to this mechanism is the novel unifying concept that the pro-asthmatic-exposed ASM can independently generate its own active glucocorticoid (i.e., cortisol), produce its own newly activated glucocorticoid receptors for the steroid, and, accordingly, use this molecular strategy to homeostatically prevent induction of the asthmatic state. This article addresses the experimental evidence that underlies the proposed homeostatic glucocorticoid signaling mechanism in ASM, followed by a discussion and depiction of the feed-forward and feedback intrinsic ASM signaling circuitry that constitutes the homeostatic state. The proposed mechanism offers a practical roadmap for future basic and translational research aimed at identifying potential key site(s) of disrupted ASM homeostasis leading to asthma.

## Introduction

From its first inception by Claude Bernard in 1854 as the “milieu interieur” to its later adaptation by Walter Cannon in 1929 into the concept of “homeostasis” (1), the latter represents the self-regulating process by which the body maintains internal stability within a narrow physiological range (i.e., “normality”) as it adjusts to disruptive internal and external influences. Thus, homeostasis is the process that involves dynamically integrated feed-forward and feedback cellular signaling events directed at maintaining bodily health, while disruption of homeostasis leads to disease (2). Although modern medicine frequently incorporates therapies effectively aimed at ameliorating the disrupted homeostatic state, such therapies are almost invariably only temporarily effective and require repeated administration and adjustment. This limitation emphasizes the need to develop true “cures” for diseases that are fundamentally based on identifying and, thereby, aimed at reversing the disrupted homeostatic state. Arguably, a large body of research conducted over the past five decades highlights airway smooth muscle (ASM) as the pivotal site of disrupted homeostasis in asthma (3, 4). Failure to date to identify the basis of this impaired ASM homeostasis largely accounts for the lack of an identifiable cure for asthma. Consequently, asthma remains the most common chronic disease in children and young adults in the developed world, despite the use of effective therapies including β2-adrenergic and corticosteroid agonists, leukotriene modifiers, and biologics.

In more recent years, major advances have been made in a host of studies investigating the intrinsic biophysical properties of ASM, and in studies examining genetic, epigenetic, immunological and inflammatory processes implicated in regulating ASM tone (3, 4). These studies have provided valuable information that has greatly expanded our knowledge of the behavior of ASM under a variety of experimental conditions. This knowledge, however, is founded on a mosaic of information that falls short of identifying the integrated physiological “big picture” underlying homeostasis in ASM. Based on a large collection of evidence derived from studies conducted in isolated ASM tissues, cultured human ASM cells, animal models of allergic asthma, and human asthmatic individuals, a heretofore unidentified homeostatic mechanism that is intrinsic to ASM and exhibited under inflammatory and non-inflammatory pro-asthmatic conditions is proposed herein. Importantly, in this context, a distinction is made from multi-cellular and systemic homeostatic control (2), and the mechanism proposed focuses on the dynamic feed-forward and feedback cellular signaling processes that are inherent to the ASM itself and that enable homeostasic regulation of its contractile responsiveness under pro-asthmatic conditions. The substantial body of published evidence that underlies the conceptual framework of the proposed intrinsic ASM homeostatic signaling circuitry is briefly presented below, followed by a discussion and schematic depiction of the integrated ASM homeostasis mechanism.

## Role of inflammatory cytokines

Many studies have demonstrated that ASM exhibits the asthmatic phenotype of increased constrictor and impaired relaxation responses when exposed to a variety of pro-inflammatory cytokines implicated in asthma (3, 4). This feature largely accounts for the presence of airway hyperreactivity and inflammation that characterizes the *in vivo* asthmatic state (5–11). Notably, apart from pro-inflammatory paracrine attraction of airway infiltrating leukocytes by ASM (12–15), the latter is capable of autologously producing and responding in an autocrine manner to a relevant number of these cytokines (e.g., IL-13, IL-4, IL-5, IL-1β) when it is sensitized under different pro-asthmatic conditions, including exposure to IgE immune complexes, superantigen stimulation, and rhinovirus inoculation, eliciting heightened contractility and impaired relaxation responses (13, 16–19).

## Role of beta2-adrenergic receptor (β2AR) desensitization

Tolerance to β2AR agonists has been demonstrated in studies examining the effects of homologous and heterologous β2AR desensitization on airway responsiveness. These studies have shown that both types of β2AR desensitization evoke the asthmatic phenotype of increased constrictor and impaired relaxation responses in isolated ASM tissues (20, 21) and cultured human ASM cells (22), as well as *in vivo* airway constrictor hyperresponsiveness in animal models of allergic asthma, together with exacerbation of the inflammatory component of the asthma phenotype (23, 24).

## Role of mitogen-activated protein kinases activation

The mitogen-activated protein kinases (MAPKs), including extracellular signal related kinase (ERK), c-JUN N-terminal kinase (JNK), and p38 MAPK, are critically involved in the signal transduction pathways that regulate physiological and pathophysiological responses. These MAPKs have been well documented as important regulators of ASM function, and previous studies have demonstrated that ERK1/2 and JNK, alone or in combination, most notably mediate the pro-asthmatic changes in ASM contractility and relaxation elicited by treatment with various pro-asthmatic inflammatory cytokines (22, 25–28), exposure to the dust mite allergen, Der p1 (29), toll-like receptor (TLR)-4 stimulation with LPS (30), as well as homologous and heterologous β2AR desensitization of ASM (20–22). Of significance, both activated (phosphorylated) ERK1/2 and JNK have been detected in ASM from airway biopsy specimens isolated from asthmatic patients, and a positive correlation was observed between the severity of asthma and the extent of phosphorylated ERK1/2 immunostaining, whereas phosphorylated p38 MAPK staining was mostly detected in the airway epithelial cells (31). In this connection, ERK1/2 activation was found to mediate the airway hyperresponsiveness and pulmonary inflammation elicited by allergen exposure in the *in vivo* atopic asthmatic state (32–34). Moreover, constitutively increased ERK1/2 activity was detected in cultured human asthmatic ASM, and this finding was attributed to upregulated activity of the βγ subunit of Gi protein and its consequent activation of the c-Src/Ras-c-Raf-MEK-ERK1/2 signaling cascade (28).

## Role of phosphodiesterase-4D (PDE4D) activation

PDE4 largely accounts for the cAMP hydrolyzing activity in smooth muscle cells (35), and the PDE4D5 variant has been detected and implicated in regulating ASM contractility (36). Accordingly, a number of studies have demonstrated a pivotal role for PDE4 activity in regulating the altered airway function and inflammation in animal models of allergic asthma (37–41), as well as in asthmatic individuals (42–44). Significantly, in this regard, PDE4D activity was found to be constitutively increased in cultured human ASM cells isolated from asthmatic patients (28, 45), and this upregulated PDE4D activity was attributed to ERK1/2 activation (28). This evidence complements that previously demonstrating that ERK1/2-mediated increased PDE4D activity is exhibited in IL-13-exposed and in β2AR-desensitized ASM tissues, and is responsible for their pro-asthmatic changes in constrictor and relaxant responsiveness (20–22). Of note, the mechanism of action of ERK1/2 in ASM was found to involve both its direct acute activation of PDE4D activity (28) and transcriptional upregulation of PDE4D transcripts *via* phosphorylation of the CREB and ATF1 transcription factors (20).

## Regulation of endogenous glucocorticoid activation

Endogenous glucocorticoids (GCs) regulate a host of critical physiological functions including inflammatory responses, cellular growth and differentiation, and various metabolic processes. Tissue bioavailability of endogenous GCs is regulated by the actions of the intracellular GC-activating and -inactivating enzymes, 11β hydroxysteroid dehydrogenase-1 (11β-HSD)1 and 11β-HSD2, respectively, that act by interconverting inert and bioactive endogenous GCs (46, 47). Accordingly, to enable endogenous GC action at an affected (e.g., inflammatory) tissue site, the circulating inert GC, cortisone, which is normally present in the tissue’s microenvironment, is converted by the intracellular oxoreductase activity of 11β-HSD1 into the bioactive derivative, cortisol, rendering the latter activated GC available to the affected tissue (47). This localized tissue self-regulating mechanism of endogenous GC activation was identified in human ASM wherein the pro-inflammatory/asthmatic cytokines, IL-13 and IL-4, were found to upregulate 11β-HSD1 expression secondary to activation of the transcription factor, AP-1 by ERK1/2 and JNK MAPK signaling (27). Notably, in contrast to 11β-HSD1, the dehydrogenase activity of 11β-HSD2 that inactivates cortisol by its conversion to cortisone was suppressed in ASM by IL-13, an effect that served to amplify the bioavailability of cortisol attributed to upregulated 11β HSD1 activity (27). Importantly, this intrinsic ASM homeostatic regulation of endogenous GC bioavailability in the cytokine-exposed state was shown to enable physiologically relevant concentrations of circulating cortisone to protect ASM tissues from the pro-asthmatic effects of IL-13 on ASM constrictor and relaxation responsiveness (27).

## Regulation of unligated glucocorticoid receptor activation

GCs act by binding to the intracellular glucocorticoid receptor (GR), thereby enabling it to dissociate from its chaperone proteins, become phosphorylated, and translocate as a homodimer to the nucleus. The intra-nuclear ligand (i.e., GC)-activated GR can then regulate gene transcription by interacting with GC response elements (GREs) residing in the promotor region of GC-responsive genes (48). Consistent with previous reports demonstrating cytokine-induced non-ligand-dependent GR activation in other cell types (49), exposure of human ASM cells to the pro-asthmatic cytokine, IL-13 (also IL-1β+TNF-α), was found to elicit ligand-independent (i.e., unbound GC) GR activation associated with nuclear translocation attributed to ERK1/2- and JNK-mediated serine site-specific GR phosphorylation (50). This cytokine-mediated ligand-independent GR activation (i.e., GR “priming”) was shown to confer heightened GC ligand-induced GR signaling in ASM resulting in enhanced GR-regulated transcriptional activity (50). Thus, these findings demonstrated that pro-asthmatic cytokine-induced ligand-independent GR “priming” serves to homeostatically amplify GC responsiveness in pro-asthmatic cytokine-exposed ASM.

## Role of GC-regulated mitogen-activated protein kinase phosphatase 1

Dual specificity mitogen-activated protein kinase phosphatase-1 (i.e., MAPK phosphatase-1 (MKP-1)), which represents the archetype of GC-induced immediate-early response genes, plays a pivotal role in suppressing inflammation by dephosphorylating MAPKs and, thereby, inhibiting their pro-inflammatory signaling (51). Accordingly, MKP-1 serves a crucial negative feedback role in regulating airway inflammation (52). Comparably, MKP-1 was found to regulate ASM function (53, 54), including the pro-asthmatic changes in ASM constrictor and relaxation responsiveness accompanying ERK1/2-induced upregulation of PDE4D following homologous β2AR desensitization (55). In this context, the ERK1/2-regulated PDE4D-induced changes in ASM responsiveness were prevented by treatment with the GC, dexamethasone, and the latter exerted its protective effect by the upregulated expression and action of MKP-1 (55). These findings highlight the fundamental role attributed to GC-induced MKP-1 expression in mediating ASM homeostasis by feedback suppression of MAPK-induced pro-asthmatic changes in ASM responsiveness. MKP-1 expression may also be further enhanced by its mRNA stabilization attributed to p38 MAPK activation, as reported in ASM treated with either TNFα (56) or sphingosine 1-phosphate (57). These findings concur with previous circumstantial evidence demonstrating that, contrasting the suppressive effect of ERK1/2 inhibition, the pro-asthmatic heightened contractile responsiveness in ASM exposed to the TLR-4 agonist, LPS, and the dust mite allergen, Der p1, both implicated in significant p38 MAPK activation, is augmented by inhibition of p38 MAPK (29, 30). Together, these observations are consistent with the notion that the negative feedback (i.e., bronchoprotective action) attributed to GC-induced upregulation of MKP-1 may be facilitated in ASM by p38 MAPK activation, resulting in increased overall GC-induced feedback inhibition of MAPKs and their downstream effects.

## The pro-asthmatic ASM homeostasis mechanism

**Figure 1:**
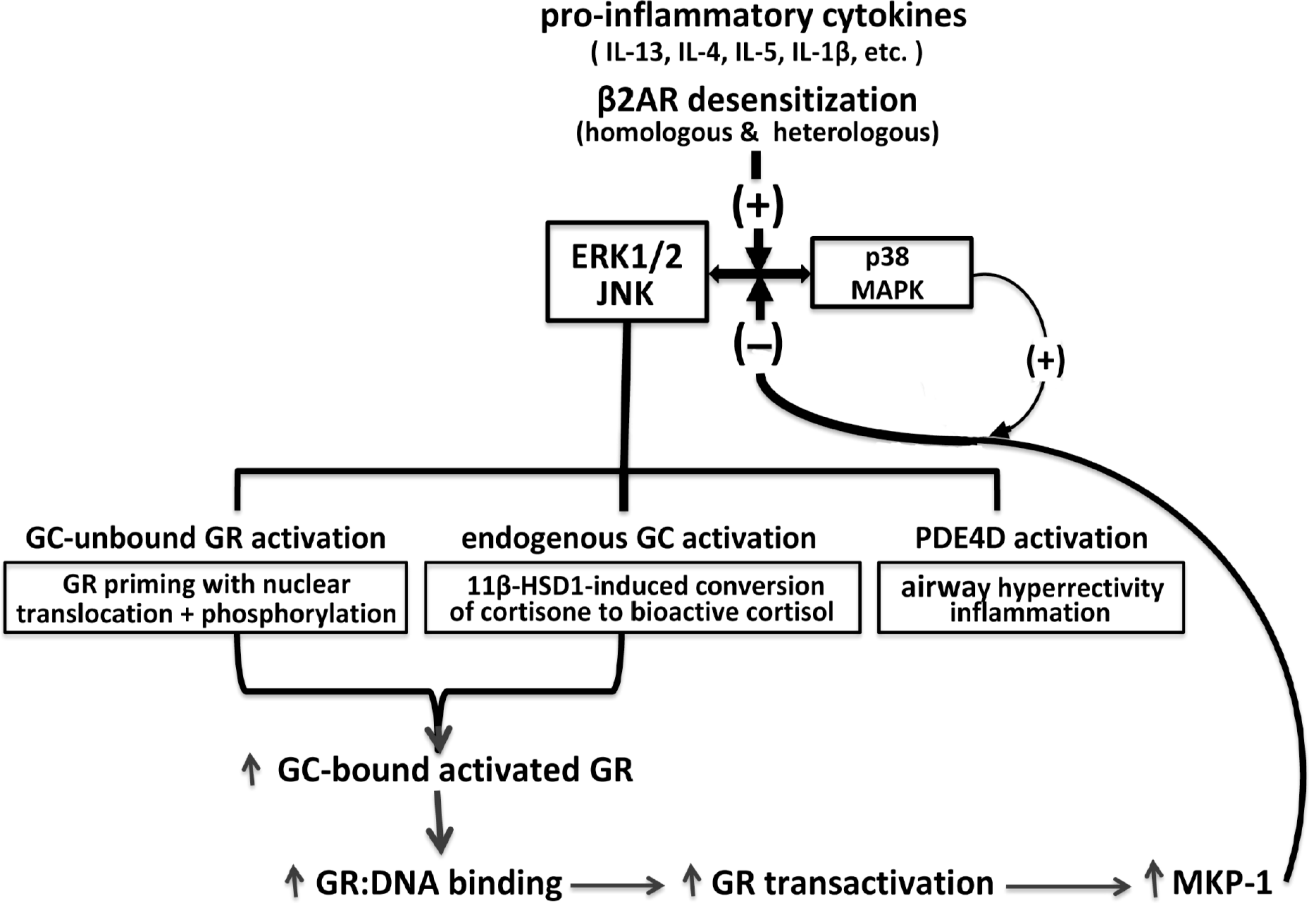
Homeostatic glucocorticoid signaling mechanism in pro-asthmatic ASM. Exposure of ASM to pro-inflammatory cytokines implicated in asthma and β2AR desensitization of ASM evokes MAPK activation. The MAPKs, notably involving ERK1/2 and JNK, elicit opposing downstream physiological events, including PDE4D-induced pro-asthmatic airway hyperreactivity and inflammation and bronchoprotective endogenous GC signaling. The latter involves 11β-HSD1-induced upregulated conversion of circulating inert cortisone into bioactive cortisol, and activation of GC-unbound GR “priming,” given by nuclear translocation with phosphorylation of the unliganded GR. Together, the latter MAPK-regulated endogenous GC actions are complementary and lead to amplified GC-bound GR activation resulting in transcriptional upregulation of MKP-1, potentially facilitated by p38 MAPK activity, resulting in feedback inhibition of MAPK signaling. At equilibrium, the MAPK-regulated feed-forward (i.e., pro-asthmatic) and feedback (i.e., GC-mediated bronchoprotective) signaling events dynamically maintain ASM homeostasis under pro-asthmatic conditions.

The above broad collection of previously published studies provides the conceptual foundation for a herein-described intrinsic homeostasis mechanism in ASM that serves to regulate its responsiveness under pro-asthmatic conditions. This proposed mechanism is schematically depicted in **Figure 1** wherein, as with activated MAPK activity that is constitutively expressed in human asthmatic ASM (28, 31), exposure of naïve ASM to a number of pro-inflammatory/asthmatic cytokines and to β2AR desensitization evokes MAPK activation (20–22, 25–28). The MAPKs (notably ERK1/2 and JNK) are responsible for activating three fundamental limbs of cellular activity that underlie the ASM intrinsic homeostasis mechanism, including: 1) pro-asthmatic signaling attributed to upregulated PDE4D-induced airway hyperresponsiveness and inflammation; 2) induction of endogenous bioactive GC production by upregulating 11β-HSD1 and its conversion of inert cortisone into bioactive cortisol, a phenomenon shown to generate a sufficient amount of cortisol by IL-13-exposed ASM from physiologically relevant circulating levels of cortisone, thereby, enabling its protection from the pro-asthmatic effects of the cytokine on ASM responsiveness (27); and 3) ligand-independent (i.e., GC-unbound) activation of GR involving its priming by nuclear translocation with phosphorylation (50). Importantly, the latter two processes are complementary, and together serve to amplify GC ligand-induced GR activation. This leads to increased GR : DNA binding activity eliciting heightened transactivation that evokes upregulated expression of MKP-1, which is potentially facilitated by temporal activation of p38 MAPK (56, 57), resulting in augmented feedback inhibition of MAPK signaling and its pro-asthmatic effects (55). Collectively, the above three limbs of physiological activity evoked by pro-asthmatic stimulation of ASM incorporate both dynamic feed-forward and feedback MAPK-regulated signaling events that, at equilibrium, constitute a dynamically maintained “closed” physiological circuit. Closure of this circuit at convergence of feed-forward and feedback MAPK regulation establishes the ASM homeostatic state. Accordingly, all three limbs of signaling become effectively “silenced” by being dynamically maintained in equilibrium within a physiologically normalized state. In this context, disruption of the circuit at any of its regulatory sites compromises ASM homeostasis and, thereby, enables a pathophysiological state of sustained pro-asthmatic ASM dysfunction. Among other possibilities, such disruptive processes might include those that interfere with homeostatically appropriate qualitative and/or quantitative changes in endogenous GC production and/or GR activation.

Central to the above mechanistic scenario is the novel unifying concept that the pro-asthmatic-stimulated ASM can independently generate its own active glucocorticoid (i.e., cortisol), produce its own newly activated glucocorticoid receptors for the steroid and, accordingly, use this self-regulated glucocorticoid molecular strategy to homeostatically prevent induction by the cytokine of the asthmatic state (i.e., *via* glucocorticoid-induced upregulation of MKP-1). Arguably, failure of this strategy of intrinsic bronchoprotection involving endogenous glucocorticoid activation and signaling by the pro-asthmatic-challenged ASM may account for the conventional need to use exogenously administered glucocorticoids in the clinical setting to substitute, at least in part, for disrupted endogenous glucocorticoid-mediated ASM homeostasis in asthmatic individuals.

While the above proposed mechanism of ASM homeostasis is built on the foundation of a broad collection of published studies, it must be emphasized that consideration should also be given to potential other intrinsic ASM, airway intercellular, inflammatory, and systemic disrupted signaling events that may importantly contribute to the overall pathobiology of impaired airway homeostasis. In the interim, it is believed that the herein described self-regulating ASM homeostatic glucocorticoid signaling mechanism serves to significantly elucidate the “puzzle” of airway homeostasis as it relates to asthma. As such, the proposed mechanism may provide a practical roadmap for future basic and translational research to identify potential key site(s) of disrupted ASM homeostasis leading to asthma.

## Data availability statement

The original contributions presented in the study are included in the article/supplementary material. Further inquiries can be directed to the corresponding author.

## Author contributions

The author confirms being the sole contributor of this work and has approved it for publication.
